# Lessons From the Survival of an Extremely Preterm Neonate Despite Challenges in a Country Where Gestational Age of Viability Is 28 Weeks

**DOI:** 10.1002/ccr3.70433

**Published:** 2025-04-16

**Authors:** Elim Kwasi Ahorlu, Andrew Mpagwuni Ziblim, Abdul‐Hanan Saani Inusah, Barbara Swanzy‐Asare, Edith Kissi

**Affiliations:** ^1^ Department of Paediatrics and Child Health Pentecost Hospital Madina Ghana; ^2^ Department of Paediatrics and Child Health University of Ghana Medical Centre Legon Ghana; ^3^ Department of Anaesthesia, Intensive Care, and Pain Management University of Ghana Medical Centre Legon Ghana; ^4^ Department of Internal Medicine Upper East Regional Hospital Bolgatanga Ghana; ^5^ Department of Health Promotion, Education, and Behaviour Arnold School of Public Health, University of South Carolina Columbia South Carolina USA

**Keywords:** gestational age of viability, hypoglycemia, neonatal intensive care unit, neonatal sepsis, preterm neonates, very low birth weight

## Abstract

Managing extremely preterm neonates with very low birth weight is challenging, especially in Ghana where viability is set at 28 weeks. This case highlights the complexities of neonatal care in low‐resource settings and emphasizes the need for improvement in health systems to revise the age of viability.

**Abbreviations**
ANC: antenatal care
CPAP: continuous positive airway pressure
CRP: C‐reactive protein
FBC: full blood count
KMC: Kangaroo Mother Care
NICU: neonatal intensive care unit
WHO: World Health Organization

## 1 Introduction

According to the World Health Organization (WHO), preterm is defined as babies born alive before 37 completed weeks of pregnancy. An extremely preterm birth is one less than 28 weeks, very preterm is one that is 28 to less than 32 weeks, and moderate to late preterm is 32 to 37 weeks [1].

The care of extremely preterm neonates with very low birth weight presents unique challenges in both resource‐rich and resource‐limited settings. Globally, studies agree that babies born before 22 weeks' gestation do not survive after birth, while there is at least a 50% chance of survival for those born between 22 and 24 weeks, albeit often with complications. A higher birth weight is associated with an increased chance of survival. The higher the gestational age, the higher the chances of survival, and in developed countries, there is up to a 90% chance of survival at 26 weeks [1, 2, 3, 4, 5].

Neonates born preterm and with low birth weight are at an increased risk of infections [6]. *Enterobacter cloacae* , in particular, is associated with neonatal sepsis and has been linked to multiple outbreaks of sepsis in neonatal intensive care units (NICUs), leading to adverse outcomes in preterm infants including prolonged hospital stays, antibiotic resistance, and increased mortality rates [7, 8, 9].

In developing countries such as Ghana, chances of survival of a baby born before 37 weeks depend on birth weight, gestational age, mode of delivery, as well as the capability of the healthcare facility to provide resuscitation and intensive care. Only 28% of babies born below 28 weeks survive [10]. Birth weight in particular has a strong role to play in the likelihood of survival [11], with rates of survival of just 14.3% of those less than 1.5 kg (very low birth weight) reported in Ghana [12, 13]. NICUs have been shown to be important in improving outcomes of neonates born preterm as well as those born with complications such as low birth weight and hypoglycemia [14]. The survival of a preterm baby is dependent on a setting that has the capabilities to support the life and growth of such a baby; it is due to this that the age of viability in developing countries is set higher [15]. Managing extremely preterm neonates with very low birth weight is particularly challenging in Ghana, where the viability threshold remains at 28 weeks due to systemic constraints. This case highlights the complexities of neonatal care in resource‐limited settings, including poorly equipped NICUs, financial barriers limiting access to treatment, and delays in tertiary care referrals. We present this case to illustrate the multifaceted barriers to preterm neonatal survival, emphasize the possibility of survival below 28 weeks despite extreme challenges, and highlight the urgent need for health system improvements to enhance neonatal outcomes in low‐resource settings.

## 2 Case History/Examination

We present the case of a neonate born to an 18‐year‐old primigravida (P1A) who was admitted to Pentecost Hospital Madina for severe anemia, with no other significant comorbidities identified. The patient had received two blood transfusions during her week‐long hospitalization. The initial ultrasound performed during admission indicated the gestational age of the baby to be exactly 27 weeks. The mother had not yet initiated antenatal care (ANC), and so routine ANC investigations were conducted upon her admission.

Plans were underway for the mother's discharge the following day when she reported sudden onset of abdominal pain, leading to preterm labour. She delivered a live female baby into a chamber pot. Midwives quickly performed cord clamping, suctioning, and stimulation. The neonate's Apgar scores were recorded as 7/10 and 8/10 at 1 and 5 min, respectively, with the passage of meconium noted but no immediate urine output observed. Intramuscular vitamin K was administered, the baby was promptly wrapped in plastic foil for thermal protection, and then referred to the babies' unit of the same facility.

### 2.1 Clinical Findings and Initial Interventions

Anthropometric measurements included a birth weight of 1.0 kg, head circumference of 17 cm (below the third percentile), chest circumference of 16 cm, and full length of 29 cm (below the third percentile). The neonate was anicteric and afebrile but appeared plethoric and exhibited signs of respiratory distress. Vital signs recorded were a temperature of 36.0°C, a pulse rate of 121 beats per minute, and oxygen saturation of 91% on room air. The neonate was hypoglycaemic; blood glucose level was 1.7 mmol/L. Respiratory, cardiovascular, and abdominal examination findings were normal. The central nervous system assessment noted a good cry along with a present but weakly sustained suck reflex, weak grasp, and incomplete Moro reflex, suggesting neurological immaturity. Genital examination revealed normal female genitalia with a slightly prominent clitoris, while spine examination revealed no abnormalities. Ballard score calculated was 11, which indicates a gestational age of approximately 28 weeks (margin of error ±2 weeks).

## 3 Methods

### 3.1 Laboratory Investigations

Initial full blood count (FBC) revealed leukocytosis with a white blood cell count of 30.46 × 10^9^/L, elevated neutrophils, lymphocytes, and a hemoglobin level of 18.4 g/dL (Table 1). FBC repeated after a week of treatment showed improvement of white blood cell count to 22.1 × 10^9^/L, with normalized neutrophils and lymphocytes, but a reduced hemoglobin level of 10.5 g/dL (Table 1). Posttransfusion FBC revealed further improvement of total and differential white blood cell count and an increased hemoglobin level of 12.0 g/dL (Table 1).

**TABLE 1:** Summary of investigations.

Parameter	Unit	Day 0	Day 15	Posttransfusion	Reference range
WBC	10^9^/L	30.46	22.21	14.26	4.0–12.0
Neut #	10^9^/L	9.69	6.92	5.37	1.800–6.300
Lymph #	10^9^/L	17.78	10.53	6.06	0.100–3.200
Mon #	10^9^/L	2.83	4.21	2.51	0.100–0.600
Eos #	10^9^/L	0.06	0.5	0.31	0.04–0.40
Baso #	10^9^/L	0.10	0.06	0.01	0.000–0.060
Hgb	g/dL	18.4	10.5	12.0	11.0–17.0
Hct	%	45.0	30.1	24.5	35.0–49.0
MCV	fL	108.2	108.2	76.5	76.0–96.0
MCH	Pg	44.3	37.8	37.5	27.0–32.0
Plt	10^9^/L	214	223.0	205	150–450
CRP	mg/L	< 5.0			< 5.0
Total bilirubin	μmol/L	177.16			1.7–180
Direct bilirubin	μmol/L	11.34			0.0–10.0
Indirect bilirubin	μmol/L	165.82			3.0–200

C‐reactive protein (CRP) levels were within the normal range (< 5.0 mg/L) (Table 1). Blood culture results later revealed a positive finding for *Enterobacter cloacae* , a Gram‐negative bacillus. The organism showed resistance to various antibiotics, including cephalosporins and beta‐lactamase inhibitors, but was susceptible to carbapenems, tigecycline, and levofloxacin (Table 2). Serum bilirubin levels indicated total bilirubin of 177 μmol/L, direct bilirubin of 11.34 μmol/L, and indirect bilirubin of 165.82 μmol/L (Table 1), which were within the phototherapy threshold determined by plotting on the neonatal jaundice graph. Tables 1 and 2 show the various investigations done with their reference values.

**TABLE 2:** Blood culture antibiogram.

Antibiotic class	Antibiotic	Result
Cephalosporins	Cefuroxime	Resistant
	Ceftriaxone	Resistant
	Cefepime	Resistant
Beta‐lactamase inhibitors	Amoxicillin–clavulanic acid	Resistant
	Piperacillin/tazobactam	Resistant
Carbapenems	Ertapenem	Sensitive
	Meropenem	Sensitive
Aminoglycosides	Amikacin	Sensitive
	Gentamicin/netilmicin	Resistant
Fluoroquinolones	Ciprofloxacin	Intermediate
	Levofloxacin	Sensitive
Others	Trimethoprim/sulfamethoxazole	Resistant
	Tigecycline	Sensitive

### 3.2 Treatment

During the neonate's first day of life, crucial interventions were initiated to manage hypoglycaemia and respiratory distress. The neonate was placed in an incubator for thermal regulation. Intranasal oxygen (INO_2_) at 1 L/min was initiated to support respiratory function, alongside broad‐spectrum antibiotics: ampicillin (50 mg 12 hourly) and gentamicin (2.5 mg daily) intravenously. Hypoglycemia was managed initially with a 4 mL bolus of 10% dextrose followed by a maintenance infusion of 10% dextrose at 3 mL/h.

From Days 2 to 4, broad‐spectrum antibiotic therapy and respiratory support continued, and trophic feeding with infant formula was commenced on Day 3 to prepare the gastrointestinal tract for full feeding. Phototherapy was initiated due to jaundice noted on Day 3, with the serum bilirubin level within the phototherapy threshold.

On Day 5, treatment was transitioned to meropenem 20 mg 12‐hourly intravenously on account of persistent hypoglycemia. Subsequently, a positive blood culture for *Enterobacter cloacae* , which was also sensitive to this antibiotic, was identified, and meropenem was continued until Day 20. Kangaroo Mother Care (KMC) was attempted on Day 9 but was halted, and INO_2_ was switched to improvised continuous positive airway pressure (CPAP) due to low saturations. Orogastric tube feeding was initiated on Day 11 for improved feeding tolerance, alongside a belated initiation of IV caffeine citrate 5 mg due to financial constraints to manage apnea. By Day 17, the neonate tolerated full feeds, and micronutrient supplementation began. A repeat FBC on Day 15 indicated low hemoglobin, necessitating a packed red cell transfusion (20 mL) on Day 16 to facilitate weaning off CPAP and resolve hypoglycemic episodes by Day 16.

From Days 21 to 31, iron supplementation (5 mg daily) was given, and syrup caffeine citrate was introduced from Day 28. INO_2_ support was discontinued by Day 28, and the neonate was removed from the incubator on Day 29, demonstrating steady weight gain, full feed tolerance, and overall clinical improvement throughout this critical period of care.

## 4 Conclusions and Results

### 4.1 Outcomes

**FIGURE 1:**
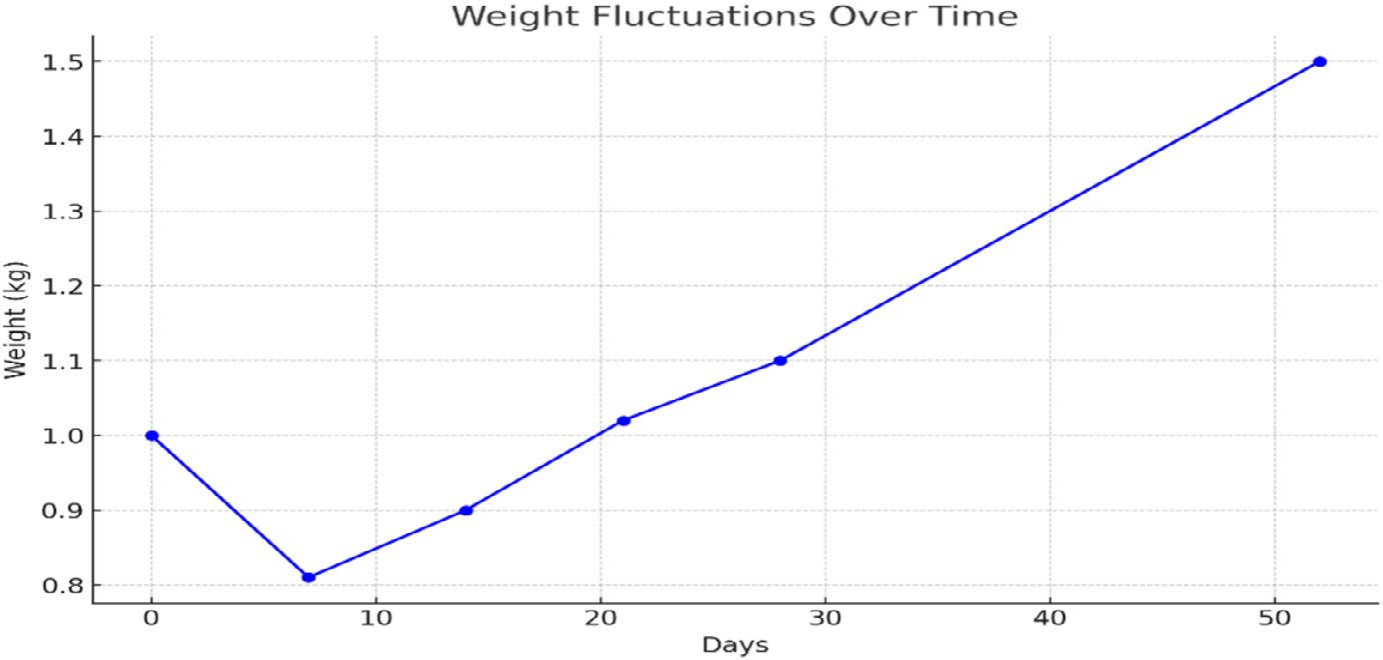
Baby's weight in kilograms over 52 days. *Source:* Patient clinical folder.

Gradual weight gain was observed after an initial drop from 1.00 to 0.81 kg by Day 7, with a subsequent steady increase to 0.90 kg by Day 14, 1.02 kg by Day 21, and 1.10 kg by Day 28 (Figure 1). Discharge weight on Day 52 was 1.5 kg, as demonstrated in Figure 1.

On discharge, the baby was saturating at 98% on room air and showed no signs of respiratory distress or sepsis. Nervous system examination was normal, and she was able to breastfeed satisfactorily.

## 5 Discussion

Extreme prematurity defined as birth before 28 weeks of gestation remains a leading cause of neonatal morbidity and mortality, particularly in low‐resource settings. These neonates are at high risk of respiratory distress syndrome (RDS) due to surfactant deficiency, hemodynamic instability, hypothermia, sepsis and feeding intolerance. In this case, the neonate exhibited respiratory distress, hypoglycemia, and poor suck reflex, consistent with extreme prematurity. Diagnosis was based on gestational age estimation, clinical presentation, and sepsis confirmation via blood culture, which identified *Enterobacter cloacae* . Management required respiratory support with intranasal oxygen and later improvised CPAP, infection control with broad‐spectrum antibiotics, and gradual enteral feeding. Delays in initiating caffeine citrate due to financial constraints and the use of improvised CPAP reflect systemic barriers that impact outcomes in such settings. Despite these challenges, the neonate achieved weight gain and clinical stability. Prognosis in extremely preterm neonates depends on gestational age, birth weight, and the availability of advanced neonatal care [15].

In high‐income countries, the age of viability is generally set between 22 and 24 weeks of gestation: these nations have well‐established NICUs equipped with state‐of‐the‐art medical technology such as advanced ventilators, incubators, and CPAP devices that can contribute to improved outcomes [15, 16]. Conversely, in low‐resource settings like Ghana, the age of viability is typically higher, around 28 weeks. The case report of a preterm neonate born at 27 weeks gestation in Ghana highlights the challenges faced in such environments, and shows the unique situation that is seen if such a birth survives the neonatal period. Even though Ghana has reasonably well‐equipped NICUs in a handful of health facilities, they are often overburdened, leading to difficulties in referral, as was encountered in this case. Literature has shown that survival of a neonate born at a gestational age below 28 weeks in sub‐Saharan Africa is possible; however, only a handful have detailed the specific challenges faced, solutions ventured, and provided a discussion in context of a revision of the gestational age of viability [10, 17]. A lot of effort was made, in spite of limitations such as lack of equipment and financial capability, to ensure the survival of this neonate. KMC was initiated early because of the well‐known benefits of skin‐to‐skin contact, especially for preterm neonates [18]. Some of these efforts are stand‐ins, such as the use of improvised methods for CPAP, and they are crucial in contributing to positive outcomes, particularly in neonates with respiratory distress in low‐resource settings [19]. The disparity of financial muscle between the developed and developing world results in poorer outcomes when it comes to preterm births [20]: in our case, caffeine citrate was not started as soon as it should have been because of lack of finances to purchase this medication, which has shown to be effective in reducing the need for longer intubation periods and mechanical ventilation, thereby reducing the risk of bronchopulmonary dysplasia, brain injury, and subsequent death [21].

One of the challenges encountered was the unexpected delivery of the neonate in a chamber pot during a preterm labor episode, highlighting the urgency and unpredictability of neonatal care in such settings as well as the greater risk of infection and sepsis faced, and this brings to fore the quality of antenatal education these mothers are receiving in low‐resource settings and whether they are fully empowered to recognize early signs of labor and know when to seek medical help [22]. The sepsis that ensued was therefore not surprising, considering the gestational age at delivery as well as the circumstances of delivery and the environment in which the child was receiving care [8]. Broad‐spectrum coverage with ampicillin and gentamicin was initiated empirically [23] but switched to meropenem due to persistent hypoglycaemic episodes and suspected sepsis, coinciding with the subsequent identification of *Enterobacter cloacae* on blood culture. This decision aligns with existing literature showing that there are multiple risk factors for neonatal sepsis, including low birth weight, prematurity, and lower socio‐economic status of the mother, and suggesting the importance of appropriate antibiotic therapy tailored to local resistance patterns and microbiological findings in neonatal sepsis [9, 24].

The challenge of maintaining adequate nutrition was notable, with trophic feeding initiated early to prime the gut, gradually transitioning to full enteral feeds alongside intravenous fluid support. Weight fluctuations observed, including a notable 19% weight loss shortly after birth, emphasize the importance of meticulous nutritional management and fluid balance in extremely preterm infants to optimize growth and improve survival rates [12]. This case report illustrates the complex challenges encountered in the management of an extremely preterm neonate born in a resource‐limited setting, highlighting critical issues such as poorly resourced NICUs, financial constraints affecting treatment access, and systemic barriers to tertiary care referral. These issues, as long as they persist, will prevent a revision of the gestational age of viability down from the current 28 weeks in Ghana. The fact that a neonate born at 27 weeks survived in spite of the challenges seen shows that there can be an improvement in health systems to ensure that babies born at less than 28 weeks have a much better chance of survival.

Although literature has shown that babies can survive even at gestational ages as low as 27 weeks in Ghana, albeit usually in tertiary facilities, this case report challenges the status quo by outlining the circumstances surrounding the birth of an extremely preterm neonate; the complications of the presentation and the resultant improvisations; and the steps taken that can serve as a reminder that urgently improved health systems can save many more lives and that a downward revision of the gestational age of viability in countries such as Ghana is long overdue. This overhaul includes an urgent need for intentional and targeted solutions such as greater investments in NICUs in peripheral health facilities to ensure equitability in all parts of the country; streamlining and improvement of the referral process to ensure that neonates in need of higher levels of care get them in a timely fashion; improvement in health insurance to reduce the need for out‐of‐pocket payments to ensure that financial constraints facing families are not a reason for delayed care; and bolstering of the healthcare force in terms of both numbers and expertise in order to contribute to the best possible outcomes of neonatal care.

Finally, more research is required in the area of outcomes of very low birth weight and extremely preterm births in developing countries in order to have an appreciation for what the reality is and what needs to be done better.

## Author Contributions

**Elim Kwasi Ahorlu:** conceptualization, data curation, investigation, methodology, writing – original draft, writing – review and editing. **Andrew Mpagwuni Ziblim:** visualization, writing – original draft, writing – review and editing. **Abdul‐Hanan Saani Inusah:** writing – review and editing. **Barbara Swanzy‐Asare:** supervision, writing – review and editing. **Edith Kissi:** writing – review and editing.

## Consent

Permission to gather data from the hospital database for the case report was obtained from the Pentecost Hospital. The study was conducted along the principles of the Declaration of Helsinki. Signed informed consent to participate was taken from the mother of the baby involved in the case report after the purpose of the report was carefully explained to the mother and she was assured of the utmost level of privacy and the fact that no identifying data was going to be included in the case report. Written informed consent for publication was obtained from the baby's mother who signed a consent form in accordance with the journal's consent policy.

## Conflicts of Interest

The authors declare no conflicts of interest.

## Data Availability

Data used in this study can be made available on reasonable request from the corresponding author.
